# Pediatric Immunodeficiency Caused by Complement Classical and Alternative Pathway Defects Due to a Homozygous CFI Variant: A Case Report

**DOI:** 10.7759/cureus.81827

**Published:** 2025-04-07

**Authors:** Jieru Wei, Cuihua Liu, Ming Tian, Guanghai Cao, Jitong Li

**Affiliations:** 1 Department of Nephrology and Rheumatology, Children’s Hospital Affiliated to Zhengzhou University, Zhengzhou, CHN

**Keywords:** complement factor i, complement pathway, genetic mutation, primary immunodeficiency, renal pathology

## Abstract

Complement factor I (CFI) deficiency is a rare primary immunodeficiency that disrupts the classical and alternative complement pathways, potentially causing severe recurrent infections and autoimmune manifestations in pediatric patients. However, the coexistence of both pathways in a pediatric patient is extremely uncommon. We report a seven-year-old patient with a rare primary immunodeficiency disorder who presented with recurrent middle ear infections, paronychia, gastrointestinal infections, and respiratory infections. Genetic testing revealed a previously unreported homozygous variant in the* CFI *gene (c.848A>G; p.D283G). Immunological testing showed a decrease in complement C3, CFI, and CFH levels in the patient. Interestingly, the patient presented with IgA vasculitis, with renal pathology showing deposits of immune complexes containing IgA, IgG, IgM, and C1q. By considering the child's condition and genetic test results, the child was treated symptomatically and received regular peritoneal dialysis treatment. Subsequently, the child's condition improved compared to before and was discharged from the hospital. This case highlights the importance of considering CFI deficiency in children with recurrent infections and abnormalities in both the classical and alternative complement pathways. Our findings expand the known phenotypic spectrum of CFI deficiency and contribute to understanding genotype-phenotype correlations in complement disorders.

## Introduction

Complement factor I (CFI) deficiency is a rare autosomal recessive disorder characterized by dysregulation of the complement cascade, leading to various immunological manifestations [1]. CFI acts as a critical regulator of both the classical and alternative complement pathways by degrading C3b and C4b [2]. Variants in the *CFI* gene can lead to uncontrolled complement activation, resulting in various clinical manifestations, including recurrent infections, kidney disease, and autoimmune disorders [1].

Recent advances in our comprehension of complement-mediated diseases have demonstrated the complexity of complement regulation and its involvement in various pathological conditions [3]. The alternative pathway, in particular, has been proven to have a substantial impact on numerous diseases, such as age-related macular degeneration and complement-mediated thrombotic microangiopathy (TMA) [4,5]. In pediatric patients, complement deficiencies present unique challenges due to their impact on developing immune systems and the potential for long-term complications. Early diagnosis and appropriate management are crucial for preventing severe complications and improving outcomes.

The present study describes a case of pediatric immunodeficiency caused by a homozygous *CFI* variant affecting both the classical and alternative complement pathways. This case is particularly interesting as it demonstrates the complex interplay between the different complement activation pathways and their role in immune defense. By presenting this case and reviewing the literature, we aim to contribute to the growing body of knowledge about complement deficiencies and their clinical manifestations in pediatric patients.

## Case presentation

A seven-year-old boy with abnormal urine test results for two and a half years. He was discovered to have kidney dysfunction for three and a half months and then was treated in the Department of Nephrology, Rheumatology, and Immunology at our hospital. The child had recurrent coughing two and a half years ago, followed by purpura on both lower limbs. At the onset of illness, a urine routine examination showed 2+ protein, leading to a diagnosis of Henoch-Schonlein purpura (HSP) and Henoch-Schonlein purpura nephritis (HSPN).

Initially, the local treatment included prednisone at doses of four tablets per day (1 mg/kg/d) and eight tablets per day (2 mg/kg/d) orally, but the urine protein did not turn negative. Later, pulse therapy with cyclophosphamide at doses of 200 mg for two days and 160 mg for two days was administered consecutively, but there was no improvement in urine protein, so it was discontinued. One year ago, the patient was treated with Chinese herbal medicine at a local traditional Chinese medicine clinic, with details unknown. During the treatment, the patient took oral *Tripterygium wilfordii *(TW) for two to three months, but the proteinuria did not improve, and steroids were gradually reduced and stopped. Three and a half months ago, the child was admitted to our hospital's intensive care unit due to "fever for eight days, abdominal pain, oliguria for five days, dark urine, two days after peritonitis surgery." Blood culture indicated a *Streptococcus pneumoniae* infection. 

During hospitalization, the child developed purulent infections in the chest, abdomen, and pelvis, with pelvic drainage fluid culture showing an *Enterococcus faecalis* infection; progressive anemia, without fragmented red blood cells; lowest platelet count of 95x10-9/L; and acute renal failure. The patient received aggressive treatment, including anti-infection therapy, blood purification, red blood cell and albumin transfusions, reduced blood pressure, and symptomatic supportive care. The patient's condition improved, with a significant decrease in infection indicators, but kidney damage persisted, presenting with 3+ proteinuria, fluctuating blood creatinine levels between 90 and 110 umol/L, gross hematuria, and no improvement in anemia. The patient was discharged, and post-discharge monitoring showed continued deterioration of kidney function, with the highest blood urea nitrogen level reaching 35 mmol/L and creatinine at 180 umol/L.

**Figure 1:**
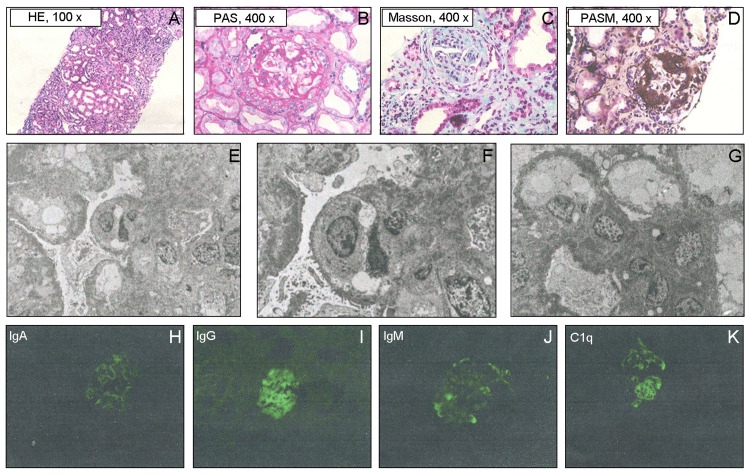
The patient's renal biopsy shows focal proliferative and sclerosing glomerulonephritis and deposition of multiple immune complexes. (A-D) The HE, PAS, PASM, and Masson staining show focal segmental intensification with endothelial cell proliferation. (E-G)Observation of ultrastructure using an electron microscope. Under the microscope, a glomerulus was detected with obvious vacuolar degeneration of the endothelial cells, focal red blood cell aggregation in some lumens, and endothelial cell proliferation. The foot processes of the glomerular basement membrane were extensively fused, showing segmental wrinkling. Mesangial cells and matrix proliferation were observed. High-density electron-dense deposits were seen in the subendothelial and mesangial areas. Segmental subepithelial electron-dense deposits were also present. (H-K) Immunofluorescence can visualize the deposition of various immune complexes such as IgA, IgG, IgM, and C1q.

The patient was diagnosed with stage 4 chronic kidney disease (CKD) upon readmission. Treatment included prednisone 1 mg/kg/d and mycophenolate mofetil dispersible tablets 20 mg/kg/d. Despite adjustments to prednisone and mycophenolate dosages, there was no improvement in proteinuria or kidney function. A kidney biopsy revealed focal segmental glomerulosclerosis (Figure 1A-1D). An electron microscope observation revealed the presence of a glomerulus with evident vacuolar degeneration in the endothelial cells, focal aggregation of red blood cells in certain lumens, and proliferation of endothelial cells. The foot processes of the glomerular basement membrane were extensively fused, exhibiting segmental wrinkling. Mesangial cells and matrix proliferation were noted, along with high-density electron-dense deposits in the subendothelial and mesangial areas. Segmental subepithelial electron-dense deposits were also detected (Figure 1E-1G). Immunofluorescence showed diffuse, granular deposits of IgG, IgM, IgA, and C1q along the capillary loops and mesangial areas (Figure 1H-1K). Other tests were negative except for decreased levels of complement factor H (CFH); CFH antibodies, CFI, C3, and C3 convertase antibodies; and a disintegrin and metalloproteinase with a thrombospondin type 1 motif, member 13 (ADAMTS13) antibodies (Table 1). The IgG concentration was 4.04-6.29 g/L (normal: 6.3-15 g/L), and the respiratory burst test was normal. The patient received aggressive treatment, including anti-infection therapy, plasma exchange, and supportive care with intravenous immunoglobulins, albumin, and plasma.

**Table 1:** Complement and antibody detection. CFH, complement factor H; CFI, complement factor I; C3, complement C3; C4, complement C4; ADAMTS-13, a disintegrin and metalloproteinase with a thrombospondin type 1 motif, member 13.

Category	Result	Reference range	Unit	Change
CFH	58.36	210-452.5	ng/mL	↓
CFH antibody	184.89	262.5-1292.5	ng/mL	↓
CFI	31.31	42.5-288.5	ng/mL	↓
C3	0.16-0.33	0.78-2.1	g/L	↓
C4	0.11-0.40	0.1-0.4	g/L	-
C3 convertase antibody	26.2	95-538	ng/mL	↓
ADAMTS13 antibody	45.05	131.25-646.5	ng/mL	↓

**Figure 2:**
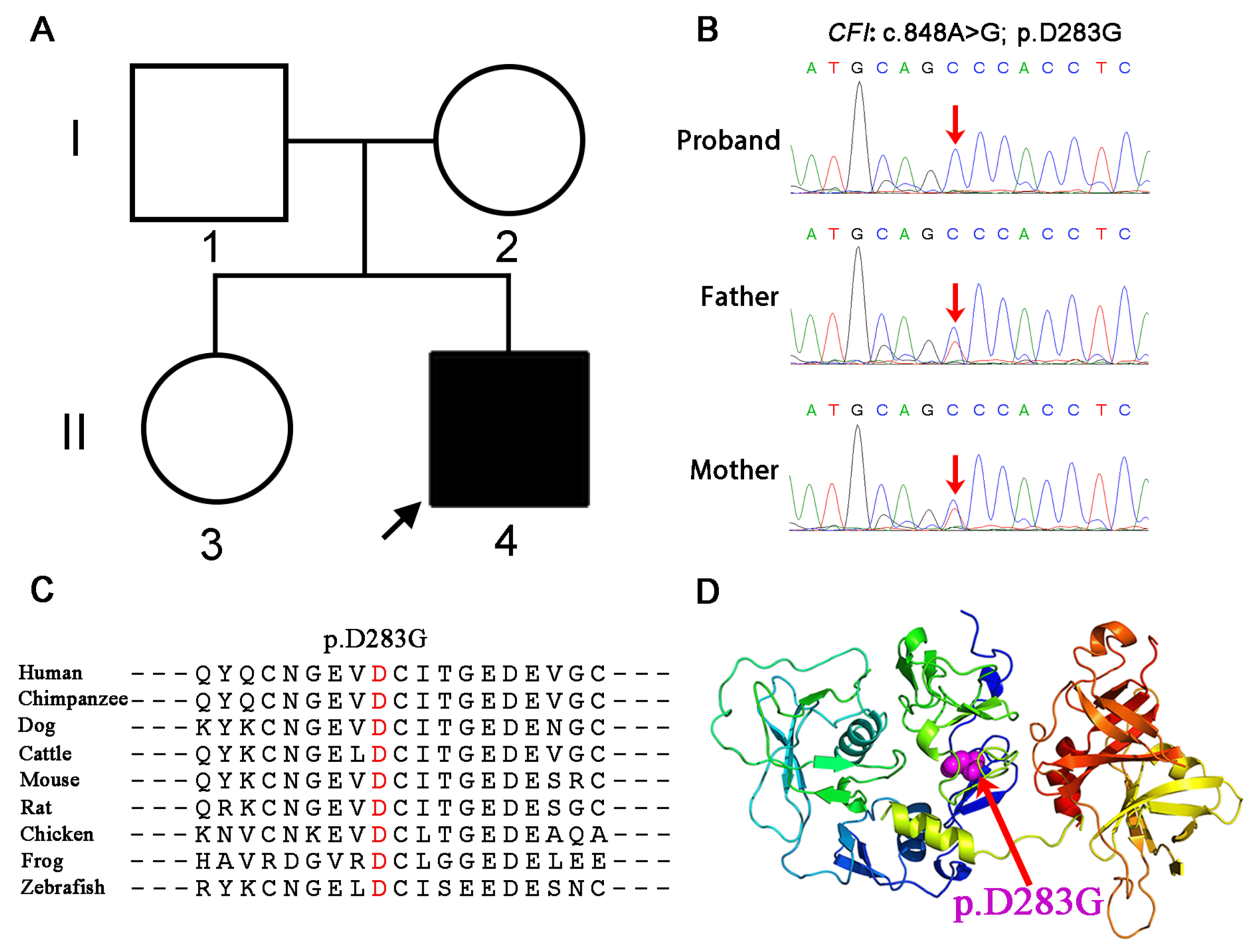
The CFI gene variant may cause protein dysfunction in the patient. (A) Family pedigree of the patient with the proband indicated by a black arrow. (B) Sanger sequencing chromatograms show the variants in *CFI* from the patient and his parents. The patient has a homozygous c.848A>G substitution in CFI (red arrow). (C) The p.D283G variant of CFI is highly conserved among various species. (D) Cartoon structural models of the human CFI protein are visualized with PyMOL software. The residue altered by the p.D283G variant is indicated in purple (as shown by the red arrow).

The whole-exome sequencing results showed a homozygous variant in the* CFI *gene (NM_000204.3:c.848A>G, p.D283G) in the patient (Figure 2B). This variant has been reported to cause C3 glomerulonephritis, although it is not currently classified as pathogenic in the ClinVar database. According to the ACMG guidelines, this variant is considered of uncertain significance. Whereas, bioinformatics protein function prediction software SIFT, PolyPhen2, and REVEL all predicted this variant to be deleterious. Family segregation analysis revealed that the parents and sister of the patient are carriers of this heterozygous variant (Figure 2A-2B). Conservation analysis across different species indicated that p.D283G is highly conserved (Figure 2C). The variant at this site could potentially alter the protein's folding and spatial conformation, thus affecting its function (Figure 2D). Based on the child's condition and genetic testing results, we stopped using MMF, gradually reduced the steroids, and regularly performed peritoneal dialysis treatment. Subsequently, the child's condition improved compared to before and has now been discharged from the hospital.

## Discussion

The complement system is an important component of the innate immune system, involved in host defense against pathogens and regulation of the body's homeostasis. The complement system can be activated through three different pathways: the classical pathway, the alternative pathway, and the lectin pathway [4]. The classical pathway is activated in the presence of immune complexes, primarily involving the activation of complement C1q. The lectin pathway is activated when mannose or ficolin recognizes carbohydrate structures. In the alternative pathway, C3 is directly activated, leading to the formation of C3 convertase and C5 convertase with the involvement of factors B, D, and P, initiating a cascade enzymatic reaction that ultimately results in the assembly of the membrane attack complex (MAC). The delicate regulation between complement activation and inactivation mediated by complement regulatory factors maintains the balance of the complement system. Any genetic or acquired factors that affect complement regulatory factors may lead to excessive complement activation and related damage.

Although the same variant (c.848A>G, p.D283G) has been described in both human and mouse models, some differences exist between the reported article and this study. Wen et al. reported a case of a 32-year-old male who developed C3 glomerulonephritis (C3GN) and TMA following a lung infection after receiving a kidney transplant. Genetic assessment revealed compound heterozygous missense variants in the *CFI *gene at c.848A>G and c.1339C>T. Zhao and colleagues reported a heterozygous *CFI* variant leading to C3 glomerulonephritis with an unknown age [6]. Song et al. found that the homozygous D288G mutants showed significant C3 deposition in the lipopolysaccharide (LPS) mouse model, indicating the involvement of the CFI D288G variant in C3 deposition [7]. In addition, Osawa et al. reported that a heterozygous *CFI* variant (c.848A>G, p.D283G) can cause C3 glomerulopathy and atypical hemolytic uremic syndrome (aHUS) in an adult patient [8]. However, in this article, we report for the first time a homozygous *CFI* variant (c.848A>G, p.D283G) occurring in a pediatric patient, and the clinical features it causes are not yet clear. The patient's CFI concentration was significantly below the normal range, leading to dysregulation of complement activation, excessive consumption of C3, and significantly decreased levels of CFB, CFH antibodies, and complement C3 convertase antibodies. Dysregulation of the complement system resulted in the patient experiencing recurrent symptoms such as otitis media, paronychia, gastrointestinal infections, and respiratory infections, consistent with the clinical symptoms of CFI immunodeficiency. Although patients with immunodeficiency caused by CFI variants are rare, there are increasing numbers of diseases associated with uncontrolled MAC activation, including aHUS, macular degeneration, and acute hemorrhagic leukoencephalitis (AHLE) [9-11].

CFI deficiency is a rare disease with a variety of clinical manifestations, including severe recurrent bacterial infections, glomerulonephritis, and systemic lupus erythematosus, with some cases of CFI deficiency being associated with aHUS [12-16]. However, the absence of TMA in this patient has led to the preliminary exclusion of aHUS. In addition, the patient's ADAMTS13 activity is normal, indicating that the patient's phenotype does not fit the thrombotic thrombocytopenic purpura (TTP) disease type.

In children with CFI deficiency, plasma levels of C1q and C4 are mostly normal [17], indicating a minor impact on the classical pathway of complement. However, in the patients in this study, C1q was found to deposit in the glomeruli, suggesting an abnormality in the classical pathway of complement. While C3 glomerulopathy is a newly emerging kidney disease caused by dysregulation of the alternative pathway of complement, characterized by C3 deposition on the glomeruli [18,19], the absence of C3 deposition in the glomeruli of the patients in this study rules out C3 glomerulopathy. It is speculated that in this case, the CFI factor may play an important regulatory role in both the classical and alternative pathways of complement. It is suggested that in CFI deficiency patients, the phenotype caused by the alternative pathway may be more severe than that caused by the classical pathway [14]. In addition, complement component deficiencies may lead to autoimmune diseases [20], vasculitis or glomerulonephritis [21]. The levels of CFH antibodies and C3 convertase antibodies were significantly decreased in the patients in this study, indicating that CFI variants may have caused abnormalities in the immune system.

Due to the patient's special condition, their response to TW may not be as effective. Research has shown that TW can alleviate proteinuria in patients with different pathological conditions and improve kidney function. Li et al. reported that combined treatment with TW polyglycosides can significantly reduce inflammation, decrease proteinuria levels, and improve renal function in patients with chronic glomerulonephritis. TW polyglycosides can enhance immune function and have a high safety profile [22]. Wang et al. reported that induction therapy with double doses of TW can significantly increase response rates in patients with severe proteinuria in IgA nephropathy without significantly increasing side effects [23]. Therefore, the efficacy of TW may vary depending on the specific pathological type. It is possible that patients with immune deficiency diseases, characterized by abnormalities in the classical and alternative complement pathways, may not respond well to TW treatment, but further research is needed.

Variants in the* CFI* gene may be associated with specific clinical phenotypes related to different CFI domains and cofactors. In children with CFI immunodeficiency, variants are commonly found in the SP, LDLRA1, SRCR, and FIMAC domains, while variants in the LDLRA2 and SEP domains are less frequent [11, 13, 17, 24]. These variants may affect protein folding in the endoplasmic reticulum (ER), leading to the retention of mutated CFI factors within the ER and preventing their secretion into the bloodstream. In the case of the c.848A>G homozygous variant causing the substitution of aspartic acid with glycine at position 283, it may result in misfolding or conformational changes in the protein, affecting the levels of CFI protein in the patient's plasma. The conservation analysis of the amino acid sequence suggests that the variant at position p.D283G may have a significant impact on CFI function.

## Conclusions

The patient was diagnosed with an immunodeficiency disorder due to a homozygous variant of c.848A>G in the *CFI* gene, which was first discovered in a child. This not only improves our comprehension of the disease but also provides the possibility for utilizing targeted therapeutic interventions based on the variants in the future. Increasing awareness of the clinical features caused by CFI and other complement deficiencies can promote early diagnosis and benefit patients through preventive treatment methods such as specific vaccine administration or prophylactic antibiotics to reduce the risk of severe infections.
